# Letter to the Editor: Author Response—The Role of Auxin in Late Stamen Development

**DOI:** 10.1093/pcp/pcaa088

**Published:** 2020-06-27

**Authors:** Ivan F Acosta

**Affiliations:** Max Planck Institute for Plant Breeding Research, Carl-von-Linné-Weg 10, 50829 Cologne, Germany

**Keywords:** Anther dehiscence, ARF6, ARF8, AUX–IAA, Auxin, Filament elongation

In our recent review on jasmonate signaling during Arabidopsis stamen maturation (Acosta and Przybyl 2019), I challenged the interpretation that Aux/IAA19 acts as a ‘key’ or ‘master’ regulator of filament elongation (Tashiro et al. 2009, Ghelli et al. 2018) and pointed out an apparent contradiction of positive and negative effects of auxin signaling in anther opening, also noticed previously (Garrett et al. 2012). I would like to respond to the letter of Cardarelli and Ghelli (2020), which presents their view on these topics and arguments against the testable models or hypotheses that I proposed in the review to reconcile the available data.

1. I agree that the exact functioning of Aux/IAA19 in filament elongation is an open question. This is precisely why I consider misleading to claim a ‘master’ regulatory role for this protein solely based on the correlated activation of *Aux/IAA19* expression and filament elongation (Ghelli et al. 2018). Without a doubt, *Aux/IAA19* expression is an excellent readout of ARF8.4 activity, but I stand by the hypothesis that it is simply part of negative feedback on ARF function, important for the switch-like behavior of auxin signaling (Lau et al. 2011).

We did not state that stamen length is similar in *massugu2/iaa19* and the single *arf6* or *arf8* mutants; instead, we clearly wrote that filament elongation is ‘delayed’ in both mutant types. This delay occurs at the critical stages 12 and 13 and is a feature shared by these and other similar weak mutants. At later stages, stamens in *arf6-2*, *arf6-101*, *arf8-1* and *arf8-3* do reach the length that wild-type stamens have at anthesis (Nagpal et al. 2005, Tashiro et al. 2009, Reeves et al. 2012). Importantly, *massugu2* is not the only auxin-resistant, (semi-)dominant mutant in an *Aux/IAA* gene showing this filament growth delay. Tashiro et al. (2009) reported a similar defect for *axr3‐1/iaa17*, and Rinaldi et al. (2012) for *iaa16-1*. According to Tashiro et al. (2009), *axr3‐1* stamens eventually elongate as much as *arf6-101* stamens, without the excess late elongation of *massugu2*.

All these (semi-)dominant *aux/iaa* mutations substitute one amino acid in the ‘degron’ motif that is essential for Aux/IAA degradation in response to auxin (Rinaldi et al. 2012). Thus, the mutant Aux/IAA proteins are predicted or have been shown to display increased stability, which normally allows stronger repression of ARFs (e.g. Gray et al. 2001). Tashiro et al. (2009) also showed delayed filament elongation in the mutant *axr1-12*, impaired in the activation of the ubiquitin-mediated proteolysis that degrades Aux/IAAs. Overall, the available genetic evidence supports that both auxin-mediated Aux/IAA degradation and ARF6/ARF8 activity are necessary for timely filament elongation. Thus, it is valid to hypothesize that ARF6 and ARF8 are targets of Aux/IAA repression.

Against this hypothesis, Cardarelli and Ghelli (2020) argue that the ARF8.4 and ARF8.2 variants are ‘defective in the region of Aux/IAA interaction’, the PB1 domain. Based on the literature alone, I do not believe that this claim is accurate because Ghelli et al. (2018) only showed that the predicted PB1 domain of ARF8.2 and ARF8.4 lacks the last 20 amino acids. They did not strictly test if this results in a ‘functionally defective’ PB1 domain. Based on the ARF5/ARF7 structural and biochemical data that support the current models of ARF-Aux/IAA interaction, I hypothesize that Aux/IAAs are still capable of interacting with and repressing ARF8.4 and ARF8.2. The 20-amino-acid truncation does not remove any of the residues essential for interaction, particularly the invariable lysine and the acidic motif that form the positive and negative charge interfaces, respectively (Korasick et al. 2014, Nanao et al. 2014). The truncated PB1 domain is missing the terminal β5 strand of the β sheet, the α3 helix and most of the α2 helix. Of these, only the absence of the β5 strand is likely to alter the natural β-grasp fold topology of the PB1 domain (Burroughs et al. 2007). I propose that this may reduce but not abolish Aux/IAA repression of ARF8.2 and ARF8.4; consequently, a lower auxin concentration threshold might be required to derepress these modified ARFs. This would agree with the model of ARF activation proposed in our review. It should also be noted, however, that truncated variants similar to ARF8.4 have not yet been described for ARF6.

I hypothesize that even a true *aux/iaa19* knock-out mutant will not show filament elongation phenotypes, due to the high redundancy of *Aux/IAA* genes. Moreover, as stated in our review, at least another five of these genes seem regulated by ARF6/8. Thus, the biochemical analysis of Aux/IAA and ARF6/8 interactions may be more informative.

2. I should emphasize that I did not question nor criticize the fact that excess auxin levels or signaling block anther dehiscence. There is clear evidence in several species supporting this view, and I believe that I summarized and cited it sufficiently in our review (Acosta and Przybyl 2019). However, it is also clear that this negative role of auxin in principle disagrees with the positive action of ARF6 and ARF8. Garrett et al. (2012) also noticed this inconsistency and called it a ‘paradox’. Perhaps my wording was perceived as ambiguous and created confusion, although I did use the terms ‘seem’ and ‘seemingly’ to indicate that this is only an apparent contradiction. In our review, I discussed this paradox and proposed a testable model to resolve it because it had been mostly overlooked in the literature.

I may have concluded incorrectly that Cardarelli’s work ‘implies’ that auxin signaling switches off before anther opening. Indeed, Cecchetti et al. (2013) only suggested that a decrease in auxin concentration (or auxin minimum) triggers opening. I also incorporated this idea into my model, but I further proposed that low auxin levels might actually activate ARF6/8 function to promote anther opening. Published work preceding our review had not explicitly proposed this and had mostly emphasized that high auxin levels or signaling block opening.

Cardarelli and Ghelli propose other solutions for the paradox: (i) ARF8.4 and ARF8.2 are not auxin-dependent factors, supposing that they are free of Aux/IAA repression. This hypothesis still needs formal testing, although, as discussed above, the current theoretical knowledge does not support it. (ii) Other ARFs may block anther dehiscence and, therefore, counterbalance the positive action of ARF6/8. Garrett et al. (2012) also proposed this based on anther indehiscence in the gain-of-function *ARF5 mp^abn^* mutant. However, it is important to recall that this phenotype results from the deregulation of ARF5 function due to a lack of Aux/IAA repression. This does not necessarily mean that the normal function of ARF5 is to block anther dehiscence. In fact, as cited in our review, deregulated *ARF6/8* expression also impairs anther opening.

In sum, I believe that both in our review and in this letter response, I have raised valid hypotheses, the testing of which may contribute to a clearer understanding of auxin and ARF6/8 function in stamen maturation and, eventually, of their interactions with jasmonate signaling.
